# Ferroelectric/paraelectric superlattices for energy storage

**DOI:** 10.1126/sciadv.abn4880

**Published:** 2022-08-03

**Authors:** Hugo Aramberri, Natalya S. Fedorova, Jorge Íñiguez

**Affiliations:** ^1^ Materials Research and Technology Department, Luxembourg Institute of Science and Technology, 5 Avenue des Hauts-Fourneaux, L-4362 Esch/Alzette, Luxembourg.; ^2^ Inter-Institutional Research Group Uni.lu-LIST on Ferroic Materials, 41 Rue du Brill, L-4422 Belvaux, Luxembourg.; ^3^ Department of Physics and Materials Science, University of Luxembourg, 41 Rue du Brill, L-4422 Belvaux, Luxembourg.

## Abstract

The polarization response of antiferroelectrics to electric fields is such that the materials can store large energy densities, which makes them promising candidates for energy storage applications in pulsed-power technologies. However, relatively few materials of this kind are known. Here, we consider ferroelectric/paraelectric superlattices as artificial electrostatically engineered antiferroelectrics. Specifically, using high-throughput second-principles calculations, we engineer PbTiO_3_/SrTiO_3_ superlattices to optimize their energy storage performance at room temperature (to maximize density and release efficiency) with respect to different design variables (layer thicknesses, epitaxial conditions, and stiffness of the dielectric layer). We obtain results competitive with the state-of-the-art antiferroelectric capacitors and reveal the mechanisms responsible for the optimal properties.

## Teaser

Ferroelectric/paraelectric superlattices can behave as artificial antiferroelectrics and can be optimized for energy storage.

## INTRODUCTION

**Fig. 1.**
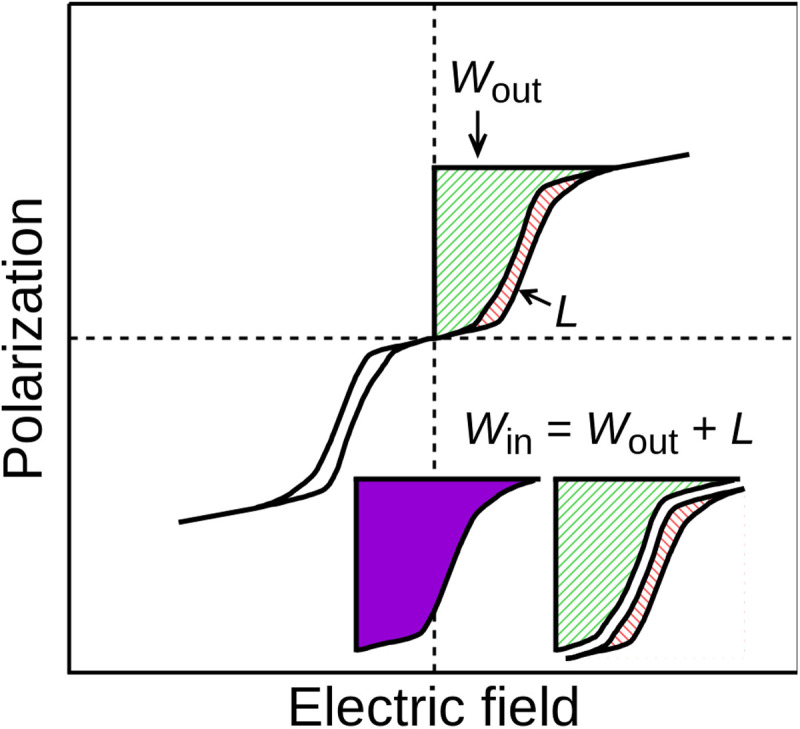
Energy storage in *P*-ε loops. The energy density required to charge the system (*W*_in_) is equal to the recovered energy density upon discharge (*W*_out_) plus the loss (*L*). Energy densities are proportional to areas in *P*-ε diagrams.

One of the limiting factors in the miniaturization of present-day electronics is the relatively large size of their capacitors, due to their somewhat low energy density. Antiferroelectric materials could help solve this problem (*1*, *2*). These compounds present an antipolar structure of electric dipoles, yielding, overall, no net polarization *P*. However, applying a large enough electric field ε can switch the system onto a polar state of large polarization (see Fig. 1). The energy density required to charge the system in this way (*W*_in_) is proportional to the area to the left of the charging branch in the *P*-ε loop, which is indicated in purple in Fig. 1. Upon removal of the electric field, the released energy density is proportional to the area to the left of the discharge branch (*W*_out_; in green in the figure). The energy loss (*L*) is therefore proportional to the area enclosed by the loop in the *P*-ε diagram (red shaded area in Fig. 1). This field-driven phase transition can be used in a capacitor with possible applications in power electronics (e.g., in electric vehicles) (*3*) and pulsed power technologies due to their fast charge/discharge rates and high energy densities (*4*, *5*). Still, relatively few antiferroelectrics are known (*2*), which hampers the optimization of the effect. Hence, there is a pressing need to discover new antiferroelectric materials.

In the past years, several efforts have been devoted to improving the energy storage performance of known antiferroelectrics. Polymers and ceramic/polymer composites can present high breakdown fields but store modest energy densities and typically suffer from poor thermal stability (*6*, *7*). Several works have reported noticeable energy densities in samples of hafnia- and zirconia-based antiferroelectrics (*8*–*12*), although with modest efficiency *W*_out_/*W*_in_ due to a strong hysteretic behavior. A more promising route explored by many groups is chemical substitution in antiferroelectric perovskites (*13*–*24*). To our best knowledge, the highest energy density in an antiferroelectric experimentally achieved to date is 154 J cm^−3^ and corresponds to a complex perovskite solid solution (*19*).

Several works have found or predicted antiferroelectricity in electrostatically frustrated perovskite oxides. Antiferroelectric phases were measured in KNbO_3_/KTaO_3_ (*25*) and SrTiO_3_/BaZrO_3_ (*26*) superlattices, although the former present scant thermal stability and the latter display antiferroelectricity for very thin layers. Theoretical works have predicted the appearance of antipolar states in BaTiO_3_/BaO superlattices (although the electric field response was not computed) (*27*) or electrostatically engineered ferroelectric thin films (which display small stability regions) (*28*). Still, the measured or predicted stability windows in these systems are rather narrow, leaving little room for optimization.

In the past years, multidomain structures have been reported in PbTiO_3_/SrTiO_3_ superlattices (*29*–*31*). The observed dipole structures can be deemed antipolar, and, hence, these systems are good candidates to display antiferroelectric-like behavior. These superlattices have attracted attention lately because they have been found to host negative capacitance (*32*), nontrivial dipole topologies (*31*, *33*, *34*), and subterahertz collective dynamics (*35*), with possible applications for voltage amplification and in electric field–driven data processing, among others.

In this work, we test the performance of ferroelectric/paraelectric superlattices as artificial antiferroelectrics for energy storage, taking PbTiO_3_/SrTiO_3_ as a relevant model system. We show that the antipolar multidomain state of these heterostructures can be switched to a monodomain polar one under an electric field, yielding response curves similar to that of Fig. 1 and thus displaying antiferroelectric-like behavior. These superlattices offer multiple design variables, including the PbTiO_3_ and SrTiO_3_ layer thicknesses, the epitaxial strain imposed by a substrate, or the stiffness of the dielectric layer (which can be controlled through its composition), which are expected to have a substantial impact in their electrostatic response and thus in their performance as capacitors. We explore these optimization possibilities by using second-principles simulation methods [which have proven successful to describe these superlattices in previous works (*31*, *32*, *36*)] to run a high-throughput investigation over these design variables. We also reveal the underlying physics yielding the best properties.

## RESULTS

**Fig. 2.**
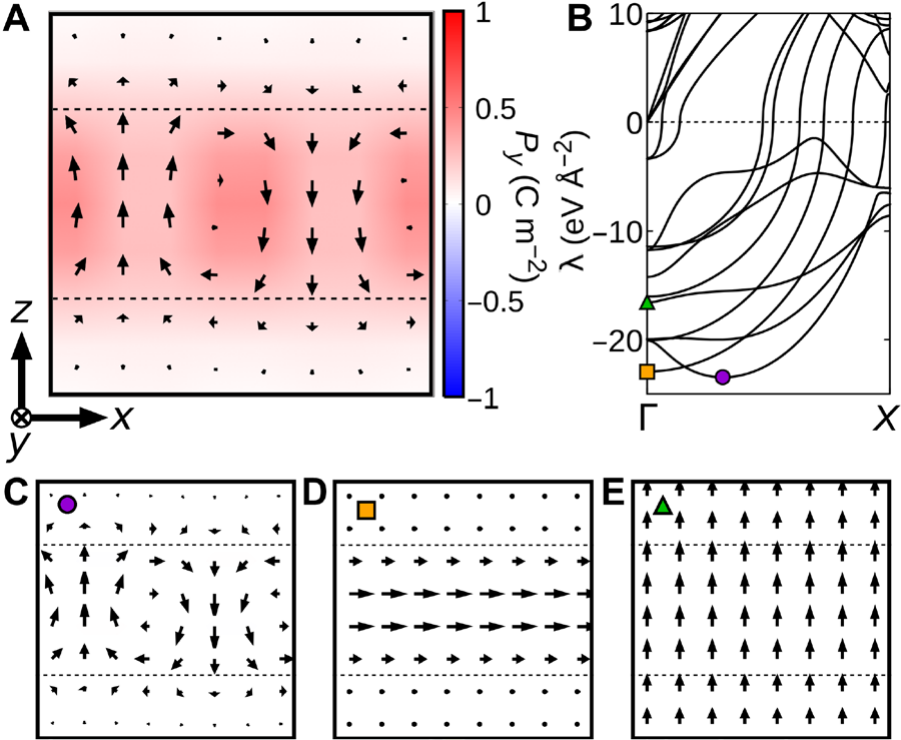
Phonon instabilities and ground state of the (PbTiO_3_)_4_/(SrTiO_3_)_4_ superlattice. (**A**) Lowest energy state of the (PbTiO_3_)_4_/(SrTiO_3_)_4_ superlattice. (**B**) Phonon instabilities of the high-symmetry (PbTiO_3_)_4_/(SrTiO_3_)_4_ superlattice (in which the atoms in the PbTiO_3_ and SrTiO_3_ layers are in the cubic phase) along the Γ-*X* direction. The eigenvectors of the leading instability (purple circle) and two relevant polar instabilities (orange square and green triangle) are shown in (**C**) to (**E**). (C to E) The eigenvectors for the phonons marked with the corresponding symbols in (B). In (A) and (C) to (E), the arrows indicate the atomic dipoles, and the out-of-screen component of the dipoles is color-coded according to the scale shown in (A).

In ferroelectric/paraelectric superlattices with the polarization easy axis along the growth direction, the development of a homogeneous polar state in the ferroelectric layer is hindered by the electrostatic penalty due to the surrounding dielectric layers. This often results in the ferroelectric component breaking into domains with opposite local polarizations, as shown in Fig. 2. The resulting state can be regarded as antipolar.

Let us consider (PbTiO_3_)*_m_*/(SrTiO_3_)*_n_* superlattices, where *m* and *n* are the thicknesses (in perovskite unit cells) of the PbTiO_3_ and SrTiO_3_ layers, respectively, and let us take the (PbTiO_3_)_4_/(SrTiO_3_)_4_ system as a representative case for the following discussion. In Fig. 2A, we show the lowest energy dipole configuration obtained for this material using second principles. It presents domains with polarization along the growth direction (*z*), yielding an overall antipolar structure. The phonon spectrum (Fig. 2B) of the high-symmetry superlattice state (in which all the atoms are fixed to the ideal high-symmetry perovskite positions) presents not only a leading antipolar instability (Fig. 2C) but also homogeneous polar instabilities with in-plane (Fig. 2D) and out-of-plane (Fig. 2E) polarizations. It is thus expected that an electric field along the stacking direction could stabilize a monodomain configuration corresponding to Fig. 2E.

This type of dipole structure was predicted by first-principles (*37*), second-principles (like those used in this work) (*32*), and phase-field (*38*) simulations and was also experimentally observed (*30*, *39*). Similar multidomain states have also been reported for BaTiO_3_/SrTiO_3_ superlattices in the past years (*40*–*42*).

**Fig. 3.**
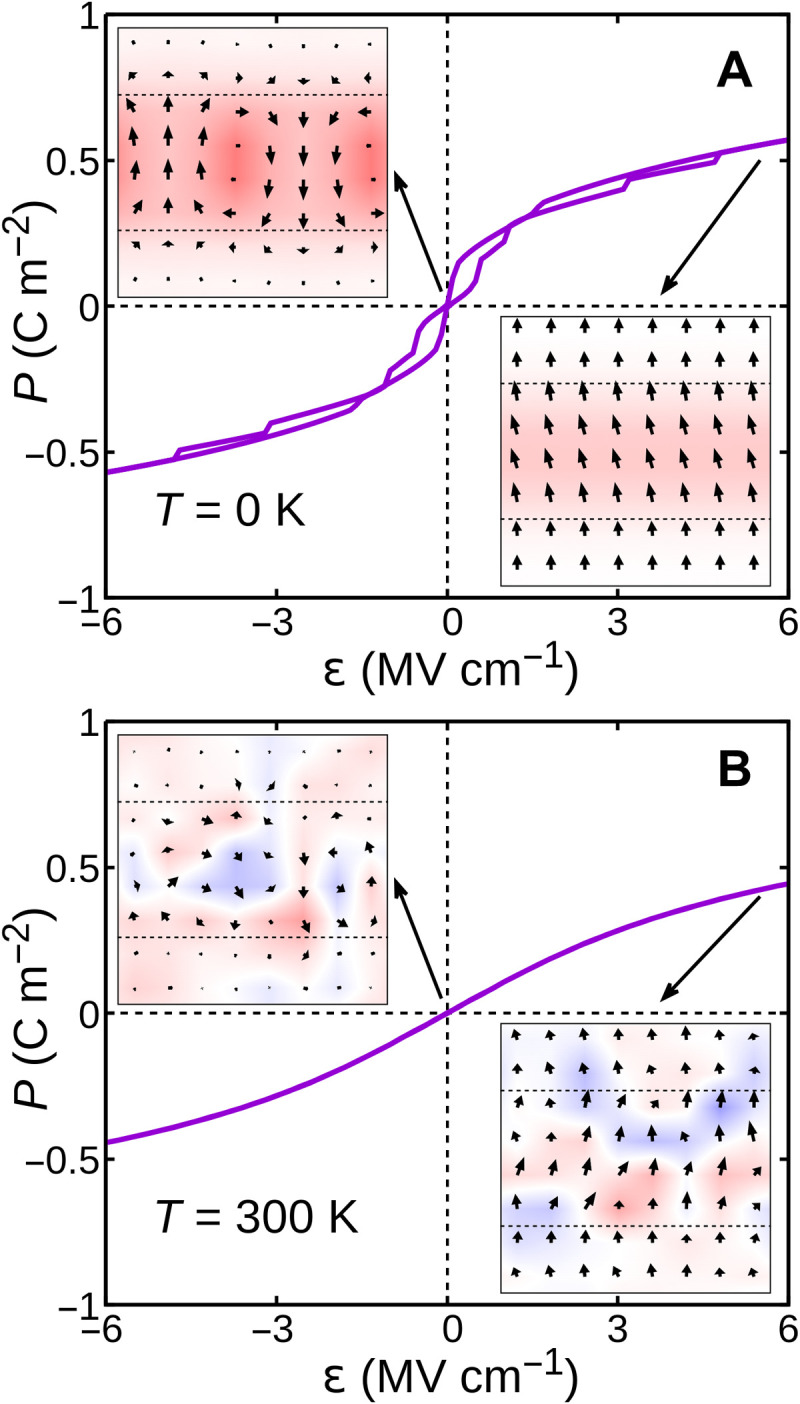
Representative *P*-ε diagrams of PbTiO_3_/SrTiO_3_ superlattices at different temperatures. (**A** and **B**) Results at 0 K and room temperature, respectively. The insets in (A) show the polarization state at zero field and at saturation. The insets in (B) show representative snapshots of the system at zero field and at saturation. The color scale for the polarization component perpendicular to the paper is that of Fig. 2A.

We make use of Monte Carlo simulations under electric field (see Methods) to compute polarization-electric field diagrams for these systems. In Fig. 3A, we show the response of the (PbTiO_3_)_4_/(SrTiO_3_)_4_ superlattice at low temperatures (strictly 0 K). The material presents the mentioned antipolar state at zero field and undergoes a field-induced phase transition onto a polar state for fields of a few megavolts per centimeter. This transition occurs in steps (corresponding to the switching of dipole columns in the ferroelectric layer) and is slightly hysteretic. Our calculations thus predict that these superlattices display antiferroelectric-like behavior.

In Fig. 3B, we show the results of a similar calculation at room temperature. It is apparent that the polarization at high fields decreases with respect to the low temperature result and that the switching is smoother and nonhysteric. Even if a static antipolar character at zero field is not apparent in instantaneous configurations (see snapshot of the system in inset to the left of Fig. 3B), the thermal average of the polarization is still zero. At large fields, the superlattice does present a clear polar order, as shown in the representative system snapshot to the right of Fig. 3B. This indicates that the antiferroelectric-like behavior is preserved at ambient conditions, which makes PbTiO_3_/SrTiO_3_ superlattices a promising playground for antiferroelectric capacitors.

We now investigate how the design parameters affect the performance of the (PbTiO_3_)*_m_*/(SrTiO_3_)*_n_* superlattices for energy storage. To this end, we run high-throughput calculations of electric field cycles up to 3.5 MV cm^−1^, with varying PbTiO_3_ and SrTiO_3_ thicknesses (between 2 and 12 and between 2 and 20 perovskite unit cells, respectively), epitaxial strain η (between −3 and +3%, taking an SrTiO_3_ substrate as the zero of the strain), and dielectric stiffness of the SrTiO_3_ layer. This stiffness can be controlled experimentally by chemical substitution, and we model it by including an additional interatomic term (denoted *h*_STO_) to favor or to penalize polar distortions in SrTiO_3_ (see Methods). In this way, we obtain a database of more than 1250 *P*-ε curves. We integrate the curves to obtain the stored energy density as a function of the maximum applied field (ε_max_), and we compute the zero-field susceptibility (χ_0_) and switching field (ε_sw_) of each *P*-ε curve (see Methods) to gain some insight into the results in terms of simple physical descriptors.

**Fig. 4.**
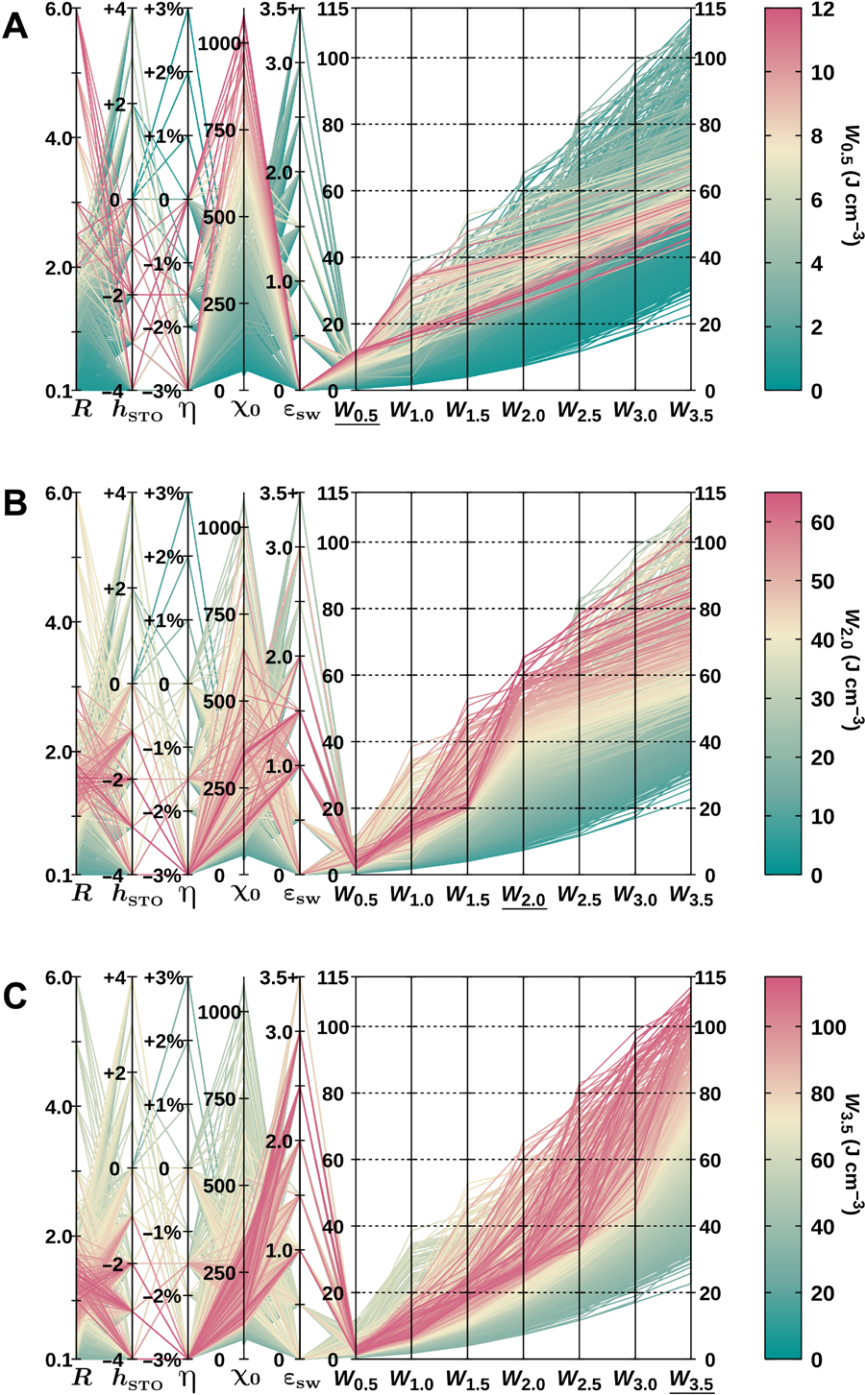
Parallel coordinates plots of the high-throughput data. (**A** to **C**) The columns, from left to right, correspond to PbTiO_3_/SrTiO_3_ ratio (*R*), modified SrTiO_3_ stiffness (*h*_STO_), epitaxial strain (η), zero-field susceptibility (χ_0_), switching field (ε_sw_), and stored energy densities at different values of the applied electric field (*W*_0.5_, *W*_1.0_, *W*_1.5_, *W*_2.0_, *W*_2.5_, *W*_3.0_, and *W*_3.5_). The lines are colored according to *W*_0.5_, *W*_2.0_, and *W*_3.5_ in (A), (B), and (C), respectively (corresponding color scales to the right of each panel).

To identify correlations, we work with parallel coordinates plots (*43*). In these plots, several vertical axes are displayed in parallel, each representing one physical descriptor. Every considered superlattice corresponds to one line in the plot, which intersects every vertical axis at the value that the superlattice takes for the attribute represented in that axis. The lines are colored according to the energy density at a given maximum field (which is also represented in one of the vertical axes), so that correlations can be visualized more easily. In Fig. 4A, we show the plot in which the color scale follows the stored energy density for a maximum applied field of 0.5 MV cm^−1^ (*W*_0.5_). By visual inspection, one can see that the best superlattices for ε_max_ = 0.5 MV cm^−1^, shown in red, are those with the largest PbTiO_3_-to-SrTiO_3_ thickness ratios *R* = *m*/*n*, the largest zero-field susceptibilities, and the smallest switching fields. It is also clear from the figure that a good performance at small applied fields essentially implies a relatively poor performance at larger fields. Even the less pronounced correlations of *W*_0.5_ with dielectrically softer SrTiO_3_ (negative *h*_STO_) and strains between 0 and −3% are also clear from the figure. Similar plots, in which the color scale represents the stored energy density at 2.0 MV cm^−1^ (*W*_2.0_) and 3.5 MV cm^−1^ (*W*_3.5_), are shown in Fig. 4 (B and C), respectively. (For plots color-coded according to the energy density at intermediate fields, see fig. S1.)

We find a strong correlation between χ_0_ and the energy density for small ε_max_ values (see Fig. 4A). Note that, in this (linear) regime, a large χ_0_ implies a large polarization response for a small applied field, which, in turn, translates into a large energy density. As the field increases (and we move into the nonlinear regime), we see that the better performing superlattices show lower values of χ_0_. A lower χ_0_ indicates a flatter initial slope in the *P*-ε diagram, which is beneficial for large enough ε_max_ values [compare, for instance, the performance of the red and green curves in Fig. 4 (A and B)].

**Fig. 5.**
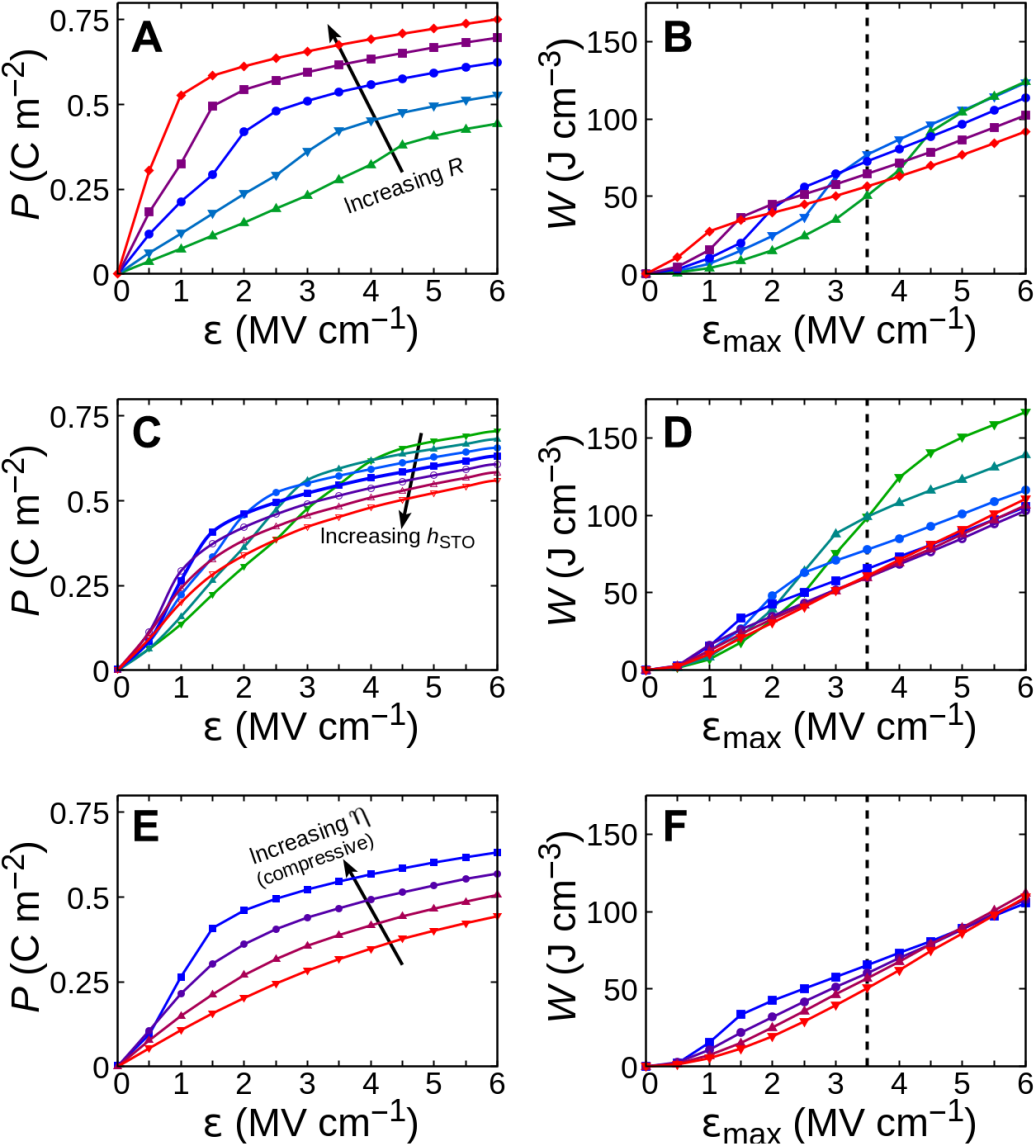
Effect of the different design parameters on the *P*-ε loops and on the stored energy density. (**A** and **B**) Variation of the *P*-ε curves and stored energy density with the PbTiO_3_-to-SrTiO_3_ ratio *R*, respectively. (**C** and **D**) Same as (A) and (B) for SrTiO_3_ stiffness. (**E** and **F**) Same as (A) and (B) for in-plane epitaxial strain η.

We observe that at low fields (Fig. 4A), a larger *R* ratio is correlated with a better performance, while at high fields (Fig. 4C), the opposite holds true. To better understand this behavior, in Fig. 5 (A and B), we show how the variation of *R* affects the polarization and energy density of representative superlattices: A thicker ferroelectric layer (or, equivalently, a thinner dielectric layer) brings the system closer to the limit of a bulk ferroelectric compound. This leads to a larger polarization in the switched state, which, in turn, increases the energy density. However, it is also apparent from these figures that this effect comes with a reduction of the switching field and that, in general, the optimal *R* will depend on the maximum applied field.

At low fields, the best performing superlattices tend to have dielectrically soft SrTiO_3_ layers (negative *h*_STO_), although many systems with unmodified stiffness (i.e., undoped SrTiO_3_; *h*_STO_ = 0) yield almost equally good performances. A correlation between softened SrTiO_3_ and high performance is apparent at medium and large applied fields. We analyze in detail the effect of varying *h*_STO_ in Fig. 5 (C and D). We see that a stiffer dielectric layer (positive *h*_STO_) imposes a larger electrostatic penalty on the polar phase and hence reduces the high-field polarization, which is detrimental for the energy density. However, it also results in a decrease in the switching field, so that (as it was the case for *R*) the optimal dielectric stiffness depends on the maximum applied field (see Fig. 5D).

A compressive epitaxial strain of up to −3% is found to be correlated with better performances in general. This is especially true for intermediate and large maximum fields, while, at low fields, the optimal strain window widens and includes unstrained superlattices. The effect of varying the epitaxial strain is illustrated in Fig. 5 (E and F). Compressive strain favors the tetragonal distortion of PbTiO_3_ and hence the alignment of its polarization along the growth direction, yielding, overall, a larger saturation polarization and increasing the energy density. At −3% strain, we observe a flattening of the initial slope in the *P*-ε curve (i.e., a decrease in χ_0_) accompanied with an increase in the switching field (from zero to a finite value). This reflects the fact that the compressive strain yields a more stable multidomain zero-field state with large local polarizations and therefore a higher energy barrier to escape out of it. Ultimately, this results in an increase in the energy density, as shown in Fig. 5F. It thus seems that, at least in the studied range of ε_max_, compressive epitaxial strain always has a positive impact on the stored energy density. This also points to the possibility of further optimizing the superlattices by using a ferroelectric layer with a larger bulk spontaneous polarization.

It is clear from Fig. 4 that the highest values of *W*_0.5_, *W*_2.0_, and *W*_3.5_ are correlated with switching fields of 0, just below 2.0 MV cm^−1^, and just below 3.5 MV cm^−1^, respectively. We overall find that, given a maximum applied field ε_max_, the stored energy density is optimal for systems that have a switching field just below the applied field $\varepsilon_{\text{sw}}\underset{\approx }{<}\varepsilon_{\text{max}}$. A late switching is beneficial for the stored energy because the area to the left of the *P*-ε curve will be larger the later the polarization develops [e.g., compare the red and green curves in Fig. 5 (A and B)]. This conclusion is in line with the ideas put forward in (*4*) and (*44*).

## DISCUSSION

The PbTiO_3_/SrTiO_3_ superlattices studied in this work present larger energy densities than most of the measured antiferroelectric capacitors. At the highest field considered, ε_max_ = 3.5 MV cm^−1^, our best superlattices store more than 110 J cm^−3^, which greatly exceeds the best results for hafnia-based antiferroelectrics (less than 40 J cm^−3^) (*8*) or relaxor ferroelectric thin films (almost 80 J cm^−3^) (*23*), while it is close to the performance of SrTiO_3_ films (about 100 J cm^−3^) (*45*) and is only surpassed by the complex perovskite solid solution (154 J cm^−3^) (*19*) that holds the present record at that field, to our best knowledge. [Note that the high-field record is held by the SrTiO_3_ films reported in (*45*), with 307 J cm^−3^ at ε_max_ = 6.6 MV cm^−1^.]

If we focus on an intermediate field of ε_max_ = 2.0 MV cm^−1^, then we find maximum energy densities of 65 J cm^−3^, which is larger than the largest value measured at that field in complex perovskite solid solutions (almost 50 J cm^−3^) (*24*), relaxor thin films (almost 30 J cm^−3^) (*46*), or hafnia-based materials (around 10 J cm^−3^) (*8*). For the lowest field considered here, ε_max_ = 0.5 MV cm^−1^, the best PbTiO_3_/SrTiO_3_ superlattice stores 12 J cm^−3^, very close to the largest experimentally observed value for perovskite solid solutions (12.6 J cm^−3^) (*24*) and outperforming the reported relaxor thin films (7.7 J cm^−3^) (*13*) and hafnia-based materials (below 2 J cm^−3^) (*8*).

Let us also note that very high energy densities have recently been predicted in AlN/ScN superlattices, up to 135 and 200 J cm^−3^ for very large fields of 5.0 and 6.3 MV cm^−1^, respectively (*47*). Still, in the cited work, these fields were rescaled by a factor of 1/3 to match an experimental response curve, so a direct comparison to our results is not possible [the fields actually considered in the simulations in (*47*) were of the order of 15 MV cm^−1^, well beyond typical breakdown fields]. Along the same lines, in (*21*), lead-free perovskite solid solutions were predicted to display energy storage performances that exceed our present results; however, electric fields were rescaled by a factor of 1/23 in that work, which complicates a direct comparison.

While the second-principles models used here have been shown to be qualitatively correct, one may wonder about their quantitative accuracy to reproduce experiments. We can get an idea by comparing our results with the *P*-ε curve of the (PbTiO_3_)_5_/(SrTiO_3_)_5_ superlattice reported in (*48*); there we find a polarization of around 0.2 C m^−2^ at 0.5 MV cm^−1^, while our simulations yield a polarization three times smaller for the same field. We attribute this to the fact that our simulated SrTiO_3_ layers are substantially stiffer than the experimental ones (*32*). At any rate, this does not affect the trends and basic quantitative results presented here. We could try to reproduce experimentally our simulated superlattices by considering Zr-doped SrTiO_3_, which should result in relatively stiff dielectric layers.

One critical aspect regarding the realization of our predictions for energy storage is yet to be addressed: the breakdown field of these superlattices. One can, in principle, compute the intrinsic breakdown field using first-principles methods (although at a high computational cost) (*49*). However, real breakdown fields are most often dictated by the presence of defects and the interfaces with the electrodes (*50*). Running realistic simulations of defects and/or capacitor/electrode interfaces comes at a notoriously high computational cost and is, in practice, unfeasible for our superlattices. Hence, this issue can only be addressed experimentally at this point. Nevertheless, let us note that there are experimental reports (*51*) of PbTiO_3_/SrTiO_3_ superlattices measured under fields of 2.38 MV cm^−1^ with no sign of breakdown, which poses a representative lower limit to the breakdown field.

In conclusion, we have computed the room-temperature energy storage capabilities of more than 1000 PbTiO_3_/SrTiO_3_ superlattices with different defining parameters. This high-throughput approach (possible thanks to the second-principles methods) allows us to identify optimal conditions, predicting that these systems outperform most of the reported antiferroelectric capacitors in a wide range of applied fields. The best materials consistently present a switching field just below the maximum applied field, indicating that tuning this variable is key to improving energy storage performance. Moreover, we find that these superlattices can be tailored to address specific needs by means of strain, layer thicknesses, and dielectric stiffness, depending on the available or desired maximum applied fields. Hence, our results indicate that electrostatically engineered ferroelectric/paraelectric superlattices are promising materials for applications in pulsed power technologies.

## METHODS

We run second-principles simulations, as implemented in the SCALE-UP code (*52*–*54*). The models for the superlattices are derived from models for bulk SrTiO_3_ and bulk PbTiO_3_ that have been used in previous works (*52*, *55*, *56*) and give correct descriptions of the lattice dynamical properties of both compounds. Then, as also described in (*31*), the interactions involving interfacial atoms in the superlattices are taken as the numerical average of the corresponding interactions in PbTiO_3_ and SrTiO_3_. To reproduce the correct long-range electrostatic behavior, an effective dielectric tensor ϵ^∞,SL^ is used for the superlattice. Along the growth direction of the superlattice, the system is considered as capacitors in series, so that the inverse of the diagonal component of the electrostatic tensor along the growth direction $\epsilon_{\mathrm{zz}}^{\infty ,\text{SL}}$ is taken as the weighted sum of the inverses of the corresponding tensor elements for bulk PbTiO_3_ ($\epsilon_{\mathrm{zz}}^{\infty ,\text{PTO}}$) and SrTiO_3_ ($\epsilon_{\mathrm{zz}}^{\infty ,\text{PTO}}$), as obtained from first principles, where the weights (*t*_PTO_ and *t*_STO_) are the relative thicknesses of the respective layers of PbTiO_3_ and SrTiO_3_

$${\left(\epsilon_{\mathrm{zz}}^{\infty ,\text{SL}}\right)}^{-1}=t_{\text{PTO}}{\left(\epsilon_{\mathrm{zz}}^{\infty ,\text{PTO}}\right)}^{-1}+t_{\text{STO}}{\left(\epsilon_{\mathrm{zz}}^{\infty ,\text{STO}}\right)}^{-1}$$ (1)

Analogously, for the in-plane components of the electrostatic tensor, the layers are considered as capacitors in parallel, resulting in an effective in-plane electrostatic tensor given by

$$\epsilon_{\mathrm{ii}}^{\infty ,\text{SL}}=t_{\text{PTO}}\epsilon_{\mathrm{ii}}^{\infty ,\text{PTO}}+t_{\text{STO}}\epsilon_{\mathrm{ii}}^{\infty ,\text{STO}}\text{for}\;i=x,y$$ (2)

Last, and to recover the correct bulk limits, the Born effective charges ($Z_{\alpha }^{*,\text{SL}}$, where α runs through the atoms) within each layer *j* are rescaled by $\sqrt{\epsilon^{\infty ,\text{SL}}/\epsilon^{\infty ,j}}$, where *j* = PTO, STO. For the Ti and O atoms at the interfaces, the Born effective charges are renormalized as follows: $Z_{\alpha }^{*}=\frac{1}{2}\sqrt{\epsilon^{\infty ,\text{SL}}/\epsilon^{\infty ,j}}Z_{\alpha }^{*,\text{PTO}}+\frac{1}{2}\sqrt{\epsilon^{\infty ,\text{SL}}/\epsilon^{\infty ,j}}Z_{\alpha }^{*,\text{STO}}$, where $Z_{\alpha }^{*,\text{PTO}}$ and $Z_{\alpha }^{*,\text{STO}}$ are the Born effective charges of atom α in bulk PbTiO_3_ and SrTiO_3_, respectively, and α = Ti, O at the interface.

The second-principles parameters of both materials are fitted from density functional theory calculations at a hydrostatic pressure of −11.2 GPa to correct for the underestimation due to the local density approximation of the cubic lattice constant that is taken as the reference structure. The dielectric stiffness of the SrTiO_3_ layer is modified by adding an extra interatomic term to the superlattice model, with the representative term (Ti*_z_*-O*_z_*)^2^, which only affects Ti and O atoms in the SrTiO_3_ subsystem. [Note that this expression is merely the representation of a symmetry-adapted term (*52*) and also affects polar distortions in SrTiO_3_ along the *x* and *y* directions.] In this way, a positive coefficient translates into an additional energy cost of polarizing the SrTiO_3_ layer (and hence the superlattice) in the growth direction. In the high-throughput calculations, the coefficient for this term, *h*_STO_, is varied between −4 and +4 meV Å^−2^.

For the ground-state calculations and the simulation of the 0-K *P*-ε diagrams, we run Monte Carlo–simulated annealings for 30,000 steps, with an initial temperature of 10 K and an annealing rate of 0.9975. To simulate electric field cycles, the studied electric field range (0 to 6 MV cm^−1^ in Figs. 3 and 5, and 0 to 3.5 MV cm^−1^ in the high-throughput calculations) is divided in increments of equal length (of 0.2 MV cm^−1^ everywhere, except for the high-throughput calculations for which an electric field step of 0.5 MV cm^−1^ was used). For each value of the electric field, a Monte Carlo simulation is performed sequentially (an annealing for the 0-K diagrams, and a constant temperature Monte Carlo simulation for the finite temperature simulations), using as initial configuration that of the previous step in the electric field ramp. To generate the finite temperature *P*-ε curves, we run Monte Carlo simulations at constant temperature for 30,000 steps at each value of the electric field, which we find to be enough to show converged results. The averages of the polarization at each value of the electric field are taken, disregarding the initial 5000 steps of each simulation, to allow for thermalization.

The high-throughput calculations are performed in a simulation cell of 8 × 2 × 1, where the unitary cell is defined as a 1 × 1 perovskite unit cell in the *xy* plane and a full superlattice period in the third direction. We check the convergence of our calculations with respect to the simulation cell. To this end, we compare the *P*-ε curves of the (PbTiO_3_)_4_/(SrTiO_3_)_4_ in simulation cells of 8 × 2 × 1, 8 × 8 × 1, and 12 × 12 × 1, both under strains of 0% and −3% (see fig. S2). We find that the results for the 8 × 8 × 1 cell are very well converged, because they are essentially identical to those of the 12 × 12 × 1 cell. The 8 × 2 × 1 cell also yields very well converged results under no strain. Under compressive strain, the switching field (inflection point of the curve) becomes finite and the results for the 8 × 2 × 1 cell are not so well converged around ε_sw_. Still, the effect is not very large, and the polarization is underestimated at lower fields, then overestimated at intermediate fields, and lastly well converged for high fields. Overall, the effect in the stored energy density is not large (specially for fields above ε_sw_).

For a given electric field ramp, the zero-field susceptibility χ_0_ is computed using finite differences. The switching field is defined as the inflection point in the *P*-ε curves. To estimate the ε_sw_ in the *P*-ε curves, we compute their second derivatives using central finite differences, and we set ε_sw_ to the largest field for which the second derivative is positive (positive curvature). In cases where the curvature of the *P*-ε curve is found to be negative for all the studied electric fields, ε_sw_ is set to zero. When the curvature is found to be positive for the full range of electric field studied (up to 3.5 MV cm^−1^), because the large field behavior has to be that of a saturating polarization with negative polarization, we set ε_sw_ to be 3.5 MV cm^−1^ or more (3.5+ in Fig. 4 and fig. S1).

The stored energy density at each value of the field is computed by trapezoid integration of the *P*-ε over the *P* axis. In the high-throughput calculations, we run a full charge-discharge cycle (from 0 to 3.5 MV cm^−1^ and then back to zero field) for several sets of design parameters (more than 50) to test for possible hysteresis, finding that none of the systems presented hysteric behavior.
