# Supplementary material for: Ferroelectric/paraelectric superlattices for energy storage
Source: Sci Adv. 2022 Aug 3;8(31):eabn4880. doi: 10.1126/sciadv.abn4880 (PMC9348786; doi:10.1126/sciadv.abn4880)
Supplement: Supplementary file 1 — Figs. S1 and S2 [file sciadv.abn4880_sm.pdf]

Science Advances
AAAS

Supplementary Materials for

Ferroelectric/paraelectric superlattices for energy storage

Hugo Aramberri *et al*.

Corresponding author: Hugo Aramberri, hugo.aramberri@list.lu

Sci. Adv. **8**, eabn4880 (2022)
DOI: 10.1126/sciadv.abn4880

The PDF file includes:

Legends for data files S1 to S8
Figs. S1 and S2

Other Supplementary Material for this manuscript includes the following:

Data files S1 to S8

Data file S1 (plain text). **Superlattice data used to create the parallel coordinates plots.** The header contains the label for each column.

Data file S2 (html format). **Interactive parallel coordinates plot of the data.** Colour code corresponds to $W_{0.5}$.

Data file S3 (html format). **Interactive parallel coordinates plot of the data.** Colour code corresponds to $W_{1.0}$.

Data file S4 (html format). **Interactive parallel coordinates plot of the data.** Colour code corresponds to $W_{1.5}$.

Data file S5 (html format). **Interactive parallel coordinates plot of the data.** Colour code corresponds to $W_{2.0}$.

Data file S6 (html format). **Interactive parallel coordinates plot of the data.** Colour code corresponds to $W_{2.5}$.

Data file S7 (html format). **Interactive parallel coordinates plot of the data.** Colour code corresponds to $W_{3.0}$.

Data file S8 (html format). **Interactive parallel coordinates plot of the data.** Colour code corresponds to $W_{3.5}$.

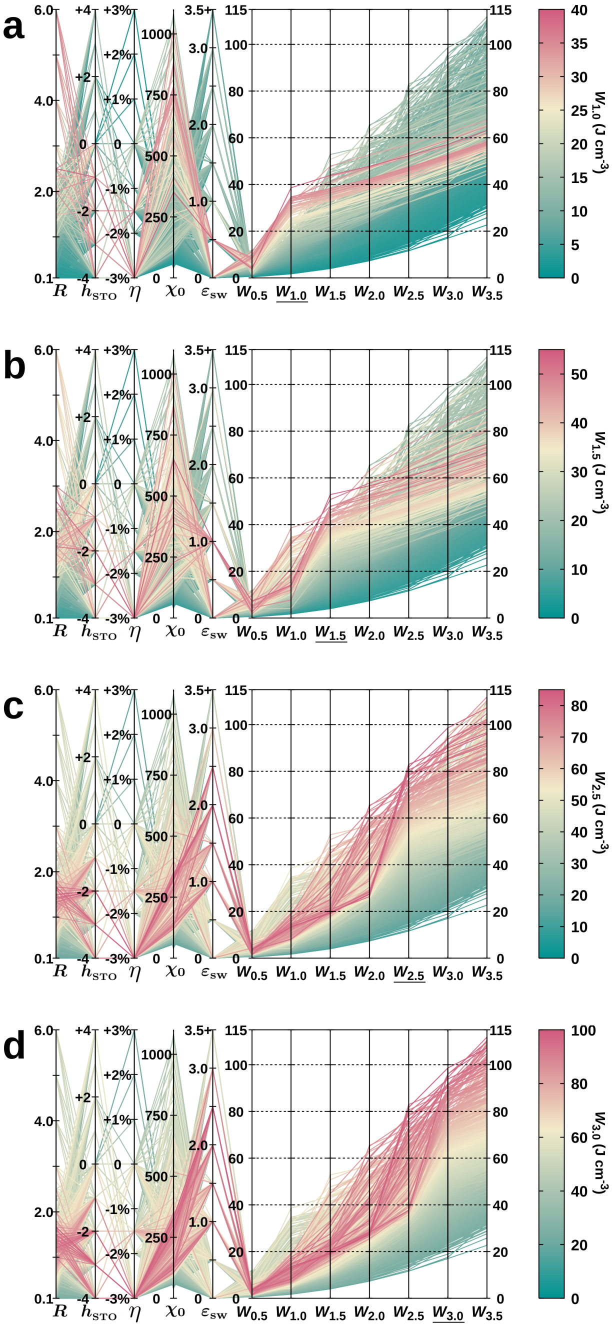

Figure S1: **Parallel coordinates plots of the high-throughput data**. The columns, from left to right, correspond respectively to $PbTiO_3$/$SrTiO_3$ ratio (R), modified $SrTiO_3$ stiffness (h_{STO}), epitaxial strain (η), zero-field susceptibility (χ_0), switching field ($\varepsilon_{\mathrm{sw}}$), and stored energy densities at different values of the applied electric field ($W_{0.5}$, $W_{1.0}$, $W_{1.5}$, $W_{2.0}$, $W_{2.5}$, $W_{3.0}$ and $W_{3.5}$). The lines are coloured according to $W_{1.0}$, $W_{1.5}$, $W_{2.5}$ and $W_{3.0}$ in panels **a**, **b**, **c** and **d**, respectively (corresponding colour scales to the right of each panel).

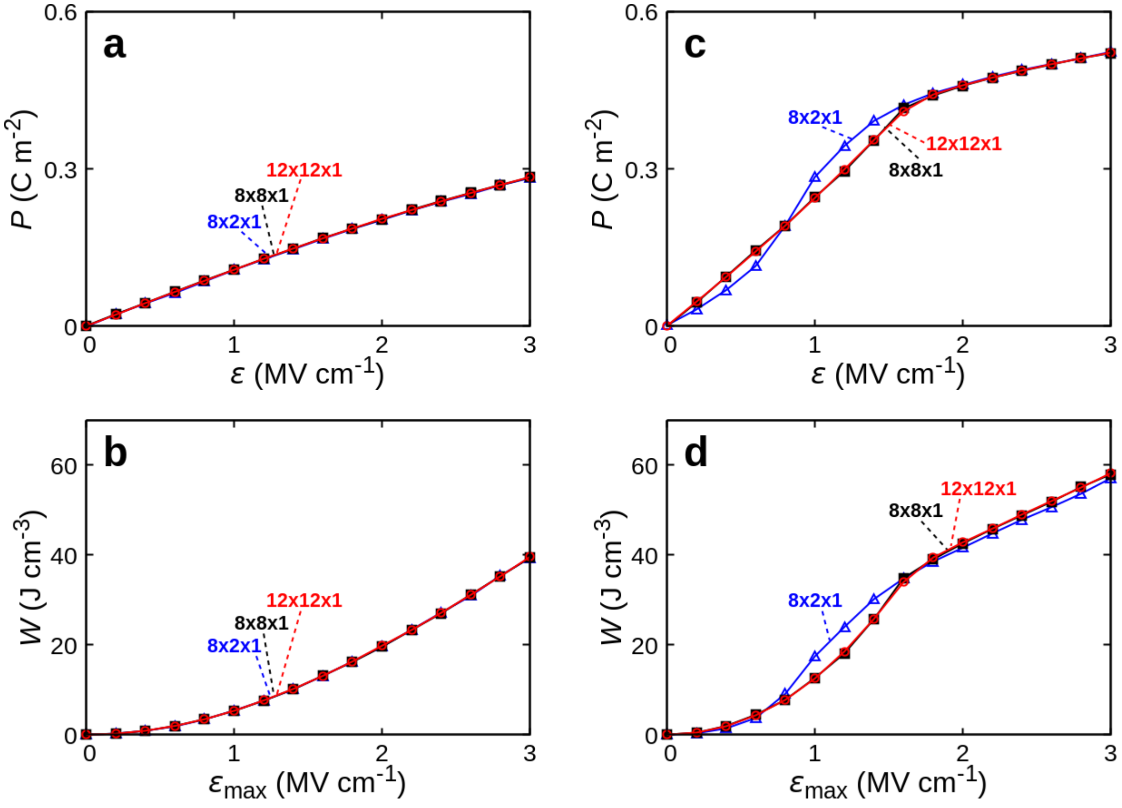

Figure S2: **Convergence of polarization response (a and c) and stored energy density (b and d) with computational cell size**. Panels **a** and **b** (**c** and **d**) show the results for a $(PbTiO_3)_4/(SrTiO_3)_4$ superlattice under 0 (-3%) epitaxial strain. The triangles, squares and circles correspond to cells of $8\times2\times1$, $8\times8\times1$ and $12\times12\times1$, respectively. The $8\times8\times1$ cell is very well converged. The $8\times2\times1$ cell employed for the high-throughput calculations in the main text is also well converged in the unstrained system. When subject to strain, the switching field is displaced to finite values and the electric field window around the switching field shows only moderate convergence, while away from the switching field the convergence remains very good.
